# Effects of temporal abiotic drivers on the dynamics of an allometric trophic network model

**DOI:** 10.1002/ece3.9928

**Published:** 2023-03-23

**Authors:** Antti P. Eloranta, Tommi Perälä, Anna Kuparinen

**Affiliations:** ^1^ Department of Biological and Environmental Science University of Jyväskylä Jyväskylä Finland

**Keywords:** abiotic forcing, biomass dynamics, food web, Lake Constance, seasonality, trophic interactions

## Abstract

Current ecological research and ecosystem management call for improved understanding of the abiotic drivers of community dynamics, including temperature effects on species interactions and biomass accumulation. Allometric trophic network (ATN) models, which simulate material (carbon) transfer in trophic networks from producers to consumers based on mass‐specific metabolic rates, provide an attractive framework to study consumer–resource interactions from organisms to ecosystems. However, the developed ATN models rarely consider temporal changes in some key abiotic drivers that affect, for example, consumer metabolism and producer growth. Here, we evaluate how temporal changes in carrying capacity and light‐dependent growth rate of producers and in temperature‐dependent mass‐specific metabolic rate of consumers affect ATN model dynamics, namely seasonal biomass accumulation, productivity, and standing stock biomass of different trophic guilds, including age‐structured fish communities. Our simulations of the pelagic Lake Constance food web indicated marked effects of temporally changing abiotic parameters on seasonal biomass accumulation of different guild groups, particularly among the lowest trophic levels (primary producers and invertebrates). While the adjustment of average irradiance had minor effect, increasing metabolic rate associated with 1–2°C temperature increase led to a marked decline of larval (0‐year age) fish biomass, but to a substantial biomass increase of 2‐ and 3‐year‐old fish that were not predated by ≥4‐year‐old top predator fish, European perch (*Perca fluviatilis*). However, when averaged across the 100 simulation years, the inclusion of seasonality in abiotic drivers caused only minor changes in standing stock biomasses and productivity of different trophic guilds. Our results demonstrate the potential of introducing seasonality in and adjusting the average values of abiotic ATN model parameters to simulate temporal fluctuations in food‐web dynamics, which is an important step in ATN model development aiming to, for example, assess potential future community‐level responses to ongoing environmental changes.

## Graphical abstract

Allometric trophic network (ATN) models provide an attractive framework to study consumer–resource interactions, but they rarely consider temporal changes in some key abiotic drivers. We investigated how seasonal changes in carrying capacity and light‐dependent growth rate of producers and temperature‐dependent mass‐specific metabolic rate of consumers affect ATN model dynamics, namely seasonal biomass accumulation, productivity, and standing stock biomass of different trophic guilds. Our findings provide novel insights into the effect of seasonality in and average value of abiotic parameters on ATN model dynamics, thus supporting further model developments for, for example, assessment of potential future community‐level responses to ongoing environmental changes.

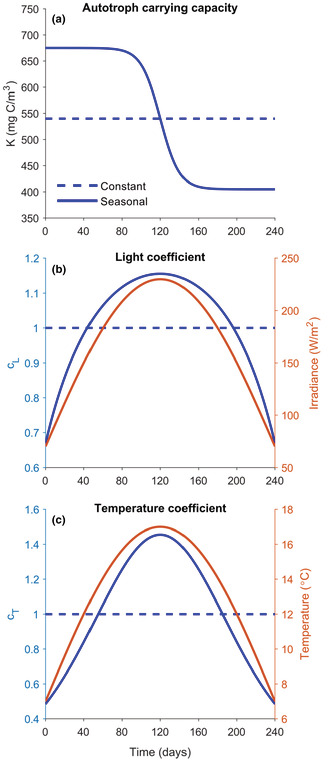

## 1 INTRODUCTION

Abiotic environmental drivers, such as temperature, light, and nutrient availability, are key factors affecting the structure, function, and productivity of terrestrial and aquatic ecosystems, with the impacts ranging across different levels of biological organization (e.g., from individual's physiology to species population density and to trophic and competitive interactions between coexisting species; Brown et al., 2004; Dunson & Travis, 1991; Gårdmark & Huss, 2020). In lakes, water temperature, nutrients, and light availability commonly shape the productivity and energy source of consumers, including zooplankton, benthic invertebrates, and fish, via complex top‐down and bottom‐up control of benthic and pelagic food‐web compartments (e.g., Shurin et al., 2012; van Dorst et al., 2020). Although these abiotic drivers are increasingly modified by climate change and other human impacts, such as land use and eutrophication (e.g., Kovalenko, 2019; Woodward et al., 2010), their effects on seasonal biomass development and standing stocks of different trophic guilds have seldom been accounted for in allometric trophic network (ATN) models (Martinez, 2020). Furthermore, the potential effects of the individual and combined abiotic drivers on ATN model dynamics have not been systematically studied.

Understanding the mechanisms of how abiotic environmental conditions influence species' abundance and interactions is critical for conservation and ecosystem management. ATN models that build on predator–prey interactions (“who eats whom”) and population dynamics depending on species' body size and metabolic type (Brose et al., 2006; Kath et al., 2018; Martinez, 2020; Williams & Martinez, 2004; Yodzis & Innes, 1992) can help to simulate ecosystem responses to environmental variation and various disturbances, such as harvesting (Kuparinen et al., 2016). The simplicity and generality of ATN models makes them attractive tools for evaluating human impacts on ecosystems, as well as for testing various ecological theories, such as coexistence theory (Brose, 2008) and biodiversity‐ecosystem functioning relationships (Schneider et al., 2016). Some parameters in ATN models, such as the growth rate of primary producers and the metabolic rate of consumers, are often set as temporally invariant constants. However, these parameters can show large seasonal and annual fluctuations and be strongly influenced by ongoing environmental changes with potential complex ecosystem‐level impacts (e.g., McMeans et al., 2015; Woolway et al., 2020). Considering temporal variation in abiotic environmental factors, such as temperature and irradiance, would likely produce ATN simulation results that better correspond with the abundance of and interactions between various trophic levels in real food webs. Factors affecting the magnitude, timing, and seasonal variation of biomass production and trophic interactions between different guilds have fundamental implications for the general ecosystem structure and function (e.g., McMeans et al., 2015) and thus should be considered also in theoretical models of community dynamics (Govaert et al., 2019; Martinez, 2020).

Indeed, the study by Boit et al. (2012) illustrates how the inclusion of abiotic forcing, that is, seasonal changes in carrying capacity, temperature, and irradiance, increases the ATN model fit to observed seasonal dynamics and size‐abundance distribution of the plankton community in Lake Constance (LC), Central Europe. However, while Boit et al. (2012) aimed to develop ATN model simulations that correspond to the observed seasonal succession of plankton community, they did not disentangle the individual and combined effects of the seasonality and average values of selected abiotic model parameters (temperature and irradiance) on the ATN model dynamics. Moreover, they did not study the potential impacts of temporally varying abiotic drivers on age‐structured fish populations in the model, namely the planktivorous European whitefish (*Coregonus lavaretus*, hereafter whitefish) and the omnivorous European perch (*Perca fluviatilis*, hereafter perch) which are the most commercially important species in the pelagic fish community of LC (Kuparinen et al., 2016).

**FIGURE 1:**
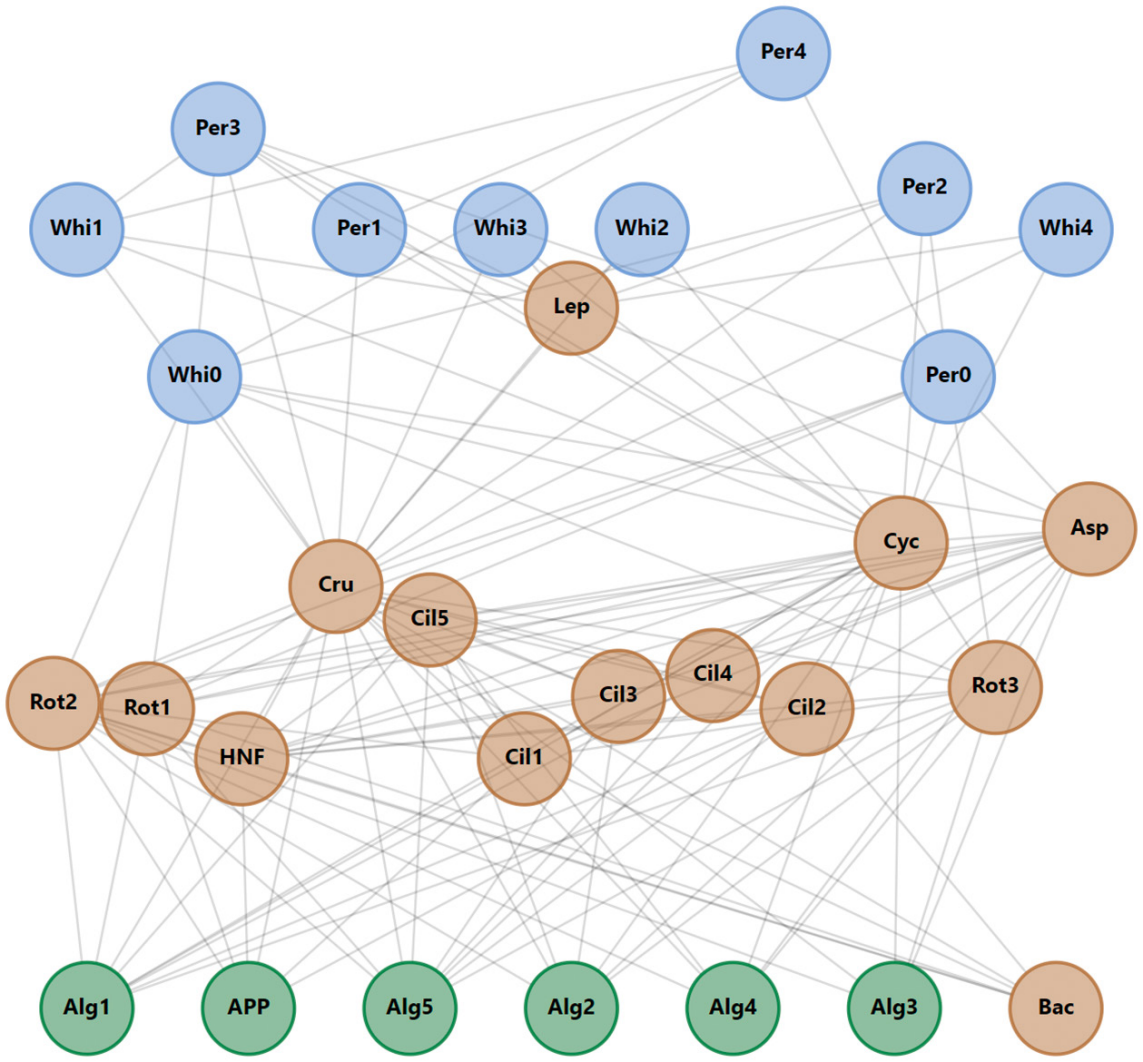
Topological structure of the simulated pelagic Lake Constance food web consisting of 133 feeding links among 30 functionally distinct guilds (i.e., species or groups of functionally similar species or fish life‐history stages), including primary producers (*n* = 6 guilds), heterotrophic microbes (*n* = 7), invertebrates (*n* = 7), and five life‐history stages of perch and whitefish (*n* = 10 guilds). See Table S1 for descriptions of the different trophic guilds.

**FIGURE 2:**
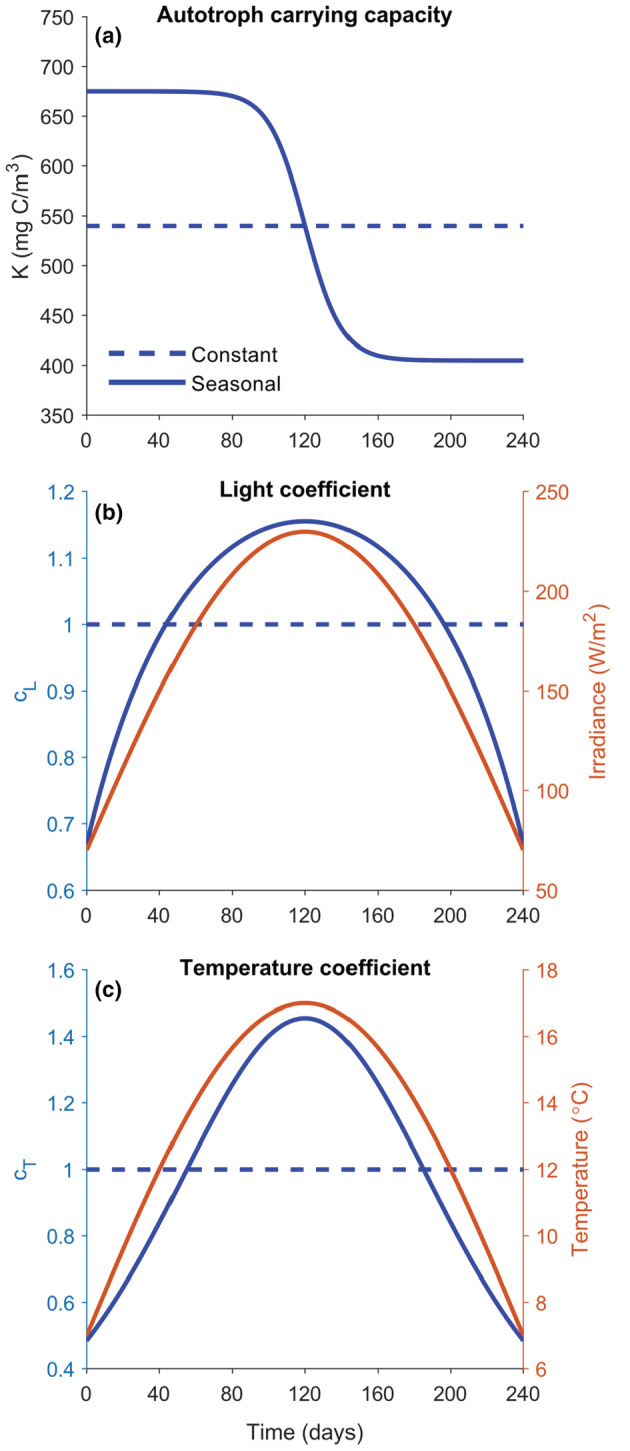
Illustrations of constant versus seasonally changing (a) autotroph carrying capacity (K), (b) light coefficient ($c_{L}$), and (c) temperature coefficient ($c_{T}$). The light coefficient $c_{L}$ affects mass‐specific growth rates of primary producers ($r_{i}$) depending on simulated average irradiance at a given day during the growing season, whereas the temperature coefficient $c_{T}$ affects the metabolic rate of consumers that is simulated to reach the highest values during the warm‐water midsummer period.

Here, we studied how linking certain key model parameters to temporal variation in selected abiotic factors affects ATN model dynamics, using an existing ATN model parametrized for the pelagic LC food web as an example study system (Figure 1; Boit et al., 2012; Kuparinen et al., 2016). More specifically, we evaluated how the inclusion of temporal variation in carrying capacity and light‐dependent growth rate of primary producers and temperature‐dependent mass‐specific metabolic rate of consumers (Figure 2) affect seasonal biomass development, productivity, and standing stock biomass of different trophic guilds, including the age‐structured populations of planktivorous whitefish and omnivorous perch. Contrary to the study by Boit et al. (2012) which aimed to simulate empirical plankton dynamics, our objective was to evaluate the individual and combined effects of the seasonality and adjusted average values of selected abiotic model parameters on the ATN model dynamics. We expected seasonally changing abiotic variables to have major effects on within‐year biomass development of primary producers and consumers and less so of top predators, that is, adult whitefish and perch. We also expected the temporal changes in producers' carrying capacity and the temperature‐dependent metabolic rate of consumers to be more important drivers of biomass dynamics than the light‐dependent growth rate of primary producers, because the former set the basal limits for secondary production and influence all consumer guilds, respectively. Despite potential changes in seasonal biomass dynamics, we expected the long‐term mean standing stock biomasses and productivity of different trophic guilds (simulated over 100 years) to be relatively robust and unresponsive to within‐year variation in abiotic drivers. To evaluate potential combined, long‐term effects of abiotic drivers that could simulate ongoing environmental changes in lakes (namely warming and reduced light penetration due to browning or eutrophication; e.g., Blanchet et al., 2022; Kritzberg et al., 2019; Woodward et al., 2010), we also investigated how the adjustment of average temperature and irradiance, as well as a gradual temperature increase of 0.037°C year^−1^ observed in LC (Adrian et al., 2009), affect the biomass dynamics of producer, invertebrate and fish guilds in the ATN model. While our simulation results should not be considered as predictions of potential seasonal and long‐term fluctuations in lake communities, they do provide important insights for future ATN model development and applications (Martinez, 2020).

## 2 MATERIALS AND METHODS

### 2.1 Lake Constance ATN model

We studied the effect of temporally varying carrying capacity of primary producers, temperature, and light availability (irradiance) on ATN model dynamics. We utilized the ATN model parametrized by the observed pelagic food‐web structure in LC (Figure 1), which has first been extended by accounting for abiotic environmental drivers (Boit et al., 2012) and further to consider age‐structured fish communities (Kuparinen et al., 2016). The modeled network consists of 133 feeding links among 30 functionally distinct guilds (i.e., species or groups of functionally similar species or fish life‐history stages), including primary producers (*n* = 6 guilds), heterotrophic microbes (*n* = 7), invertebrates (*n* = 7), and five life‐history stages of two fish species (*n* = 10 guilds; Figure 1, Table S1). The fish guilds include larvae, juveniles, 2 years, 3 years, and 4 years or older whitefish and perch. The ATN model parametrization and simulations were done in Matlab version R2021a.

The biomass dynamics of the LC food web (see Tables S1 and S2 for details) within the growing season of year Y are described by a set of ordinary differential equations (ODEs). We denote the carbon biomass density (hereafter “biomass”) of guild i by $B_{Y,i}\left(t\right)$ ($\mathrm{\mu gC}/m^{3})$ and its derivative with respect to time (days) by ${\dot{B}}_{Y,i}(t)$, where $t\in \left[t_{\mathrm{init}},t_{\mathrm{end}}\right]$. The vector of all guild biomasses is denoted by $B_{Y}\left(t\right)$. Following Boit et al. (2012), the length of the growing season is set to 240 days, and thus, we set $t_{\mathrm{init}}=0$ and $t_{\mathrm{end}}=240$. To simplify the presentation, year Y and time t are omitted from the description of the growing season dynamics. The following ODEs describe the biomass dynamics for (1) producers and (2) consumers:

(1) $$\dot{B_{i}}=\overset{\text{gain from producer growth}}{\overbrace{r_{i}B_{i}G_{i}\left(B\right)\left(1-s_{i}\right)}}-\sum_{j}^{}\overset{\text{loss to consumer}\;j}{\overbrace{\frac{x_{j}y_{\mathrm{ji}}B_{j}F_{\mathrm{ji}}\left(B\right)}{e_{\mathrm{ji}}}}}$$

(2) $$\dot{B_{i}}=-\overset{\text{maintenance loss}}{\overbrace{f_{m}x_{i}B_{i}}}+\overset{\text{gain from resources}\;\left(j\right)}{\overbrace{f_{a}x_{i}B_{i}\sum_{j}y_{\mathrm{ij}}F_{\mathrm{ij}}\left(B\right)}}-\sum_{j}^{}\overset{\text{loss to consumer}\;j}{\overbrace{\frac{x_{j}y_{\mathrm{ji}}B_{j}F_{\mathrm{ji}}\left(B\right)}{e_{\mathrm{ji}}}}}$$

where $r_{i}$ $\left({\mathrm{day}}^{-1}\right)$ is the intrinsic growth rate of producer i and it is calculated based on allometric scaling

(3) $$r_{i}=a{\left(\frac{M_{i}}{M_{0}}\right)}^{-A}$$

where the allometric scaling constant $a=1\;{\mathrm{day}}^{-1}$ and the allometric scaling exponent A=0.15, $M_{0}$ is the body mass of the reference producer guild (Alg1) and $M_{i}$ is the body mass of producer guild i, both expressed in terms of their dry carbon weight (μgC/individual; Table S1, Boit et al., 2012). $G\left(B\right)=1-\frac{1}{K}C^{*}B$ is the limiting factor in the producers' logistic growth model where 1 denotes the vector of ones and K $\left(\mathrm{\mu gC}/m^{3}\right)$ is the carrying capacity shared by all primary producers. The matrix $C^{*}=\left(1^{T}{\left(C^{T}C\right)}^{-1}C^{T}1\right)C$, where the matrix C consists of the producer competition coefficients $c_{\mathrm{ij}}$, is used to normalize the competition coefficients such that K equals the realized carrying capacity of the primary producers. Here, ${\left(∙\right)}^{T}$ denotes the matrix transpose and ${\left(∙\right)}^{-1}$ the matrix inverse. The fraction of new producer biomass lost to exudation is $s_{i}$.

The metabolic rate of consumer i is $x_{i}$ $\left({\mathrm{day}}^{-1}\right)$ and it is based on allometric scaling

(4) $$x_{i}=a{\left(\frac{M_{i}}{M_{0}}\right)}^{-A}$$

where the allometric scaling constant a and the allometric scaling exponent A are 0.314 ${\mathrm{day}}^{-1}$ and 0.15 for invertebrates and 0.88 ${\mathrm{day}}^{-1}$ and 0.11 for fish, respectively (Boit et al., 2012). The maximum consumption rate scaling factor of guild i feeding on guild j is $y_{\mathrm{ij}}$. The inefficiencies in the biomass transfer are accounted for by assimilation efficiency parameter $e_{\mathrm{ji}}$. $f_{m}$ is the maintenance respiration coefficient, and $f_{a}$ is the fraction of consumers' assimilated carbon used for production of new biomass under activity, including locomotion, foraging (food handling and digestion), ontogenetic processes, and reproduction (Boit et al., 2012; Kath et al., 2018). The normalized functional response of invertebrate and fish consumer guilds to prey guild densities is:

(5) $$F_{\mathrm{ij}}\left(B\right)=\frac{{\omega_{\mathrm{ij}}B_{j}}^{q}}{B{0_{\mathrm{ij}}}^{q}+d_{\mathrm{ij}}B_{i}{B0}_{\mathrm{ij}}^{q}+\sum_{l=\text{resources}}\omega_{\mathrm{il}}{B_{l}}^{q}}$$

where $\omega_{\mathrm{ij}}$ is the prey preference for consumer guild i feeding on resource guild j and is set to the reciprocal of the number or resources of guild i, q is the Holling exponent, $B0_{\mathrm{ij}}$ is the half‐saturation density $\left(\mathrm{\mu gC}/m^{3}\right)$ describing the biomass of the resource at which the consumer achieves half of its maximum feeding rate when consuming only resource j and in the absence of feeding interference, and $d_{\mathrm{ij}}$ $\left(m^{3}/\mathrm{\mu gC}\right)$ is the coefficient of intraspecific feeding interference. The feeding link‐specific parameters $B0_{\mathrm{ij}}$ and $d_{\mathrm{ij}}$ are determined by the type of the consumer and its resource and possibly their body mass ratio (Bland et al., 2019). The list of all aforementioned parameters, their units, value ranges, descriptions, and references is provided in Table S2, whereas the guild‐specific intrinsic growth rates and average metabolic rates are presented in Table S1.

The adult fish guilds allocate a portion of their consumed biomass to reproduction. The amount of biomass allocated depends on the total consumption gains ${ℊ}_{i}=f_{a}x_{i}B_{i}\sum_{j}y_{\mathrm{ij}}F_{\mathrm{ij}}\left(B\right)$ and the maintenance losses $\ell_{i}=f_{m}x_{i}B_{i}$. We use a piecewise defined model for the rate of biomass allocation to reproduction by adult fish guild i during the growing season:

(6) $${\dot{B}}_{i}^{+}=\left\{\begin{array}{cc} P_{i}I_{i}∙\frac{{{ℊ}_{i}}^{2}}{2\ell_{i}}, & {ℊ}_{i}<\ell_{i}\\ P_{i}I_{i}∙\left({ℊ}_{i}-\frac{1}{2}\ell_{i}\right), & {ℊ}_{i}\geq \ell_{i} \end{array}\right.,i\in \left\{25,\ldots ,30\right\}$$

where $P_{i}$ denotes the age‐dependent proportion of mature biomass in adult fish guild i, and $I_{i}$ is an age‐dependent parameter controlling reproductive investment. This model has three desirable properties: (1) it ensures that reproduction is zero when consumption gains are zero, (2) it enforces impaired reproduction when the maintenance losses are greater than the consumption gains, and (3) when the consumption gains exceed the maintenance losses, reproduction increases linearly as a function of the consumption gains. The biomass allocated to reproduction is unavailable for growth and is thus subtracted from the rate of biomass gained by consumption by adult fish guild i:

(7) $$\dot{B_{i}}=-f_{m}x_{i}B_{i}+f_{a}x_{i}B_{i}\sum_{j}y_{\mathrm{ij}}F_{\mathrm{ij}}\left(B\right)-{\dot{B}}_{i}^{+}$$

Furthermore, the amount of accumulated biomass allocated to reproduction by adult fish guild i during the growing season is solved by adding Equation 6 to the system of ODEs.

At the end of the growing season of year Y, the fish biomass is moved up one age class to become the initial biomass of the one‐year older age class for year Y+1

(8) $$B_{Y+1,\mathrm{age}+1}\left(t_{\mathrm{init}}\right)=B_{Y,\mathrm{age}}\left(t_{\mathrm{end}}\right)$$

with the exception that for the final age class of ≥4‐year‐old fish, the initial biomass is the sum of the end biomasses of the 3‐year and 4‐year‐old age classes

(9) $$B_{Y+1,4}\left(t_{\mathrm{init}}\right)=B_{Y,3}\left(t_{\mathrm{end}}\right)+B_{Y,4}\left(t_{\mathrm{end}}\right).$$

The larvae biomass (age 0) is calculated as the sum of the biomasses allocated to reproduction by all adult age classes (ages >2)

(10) $$B_{Y+1,0}\left(t_{\mathrm{init}}\right)=\sum_{\mathrm{age}=2}^{4}B_{Y,\mathrm{age}}^{+}\left(t_{\mathrm{end}}\right).$$

In addition, as whitefish does not reproduce naturally in LC, 200 units of whitefish larvae are stocked to the system at the end of each growing season. For the other guilds, biomass at the end of the growing season becomes the initial biomass for the next year's growing season.

### 2.2 Abiotic forcing in ATN model dynamics

We introduced seasonal variation in the parameters for carrying capacity (K) and mass‐specific growth rates ($r_{i}$) of primary producers and metabolic rates of consumers ($x_{i}$) to elucidate their effects on the ATN model dynamics (Figure 2; Table 1). We largely followed the approach and used the same parameter values as described in the Appendix S1 of Boit et al. (2012) but made some adjustments to the seasonal models. Essentially, we assumed that the constant model is a lower resolution approximation of the seasonally varying models that corresponds to the seasonally varying model on average in some meaningful way. Thus, we decided to normalize the seasonal models such that the average total producer biomasses are equal in each model. This way we were able to test how the seasonally varying parameter values affect the biomass dynamics. We then also tested the effects of adjusting the average values of some abiotic model parameters (i.e., temperature and irradiance).

**TABLE 1:** Overview of the parameters used in different ATN simulation scenarios.

Model name	Carrying capacity model	Autotroph growth rate model	Consumer metabolic rate model	$T_{\mathrm{adj}}$	$I_{\mathrm{adj}}$
Constant ATN	Constant	Constant	Constant	N/A	N/A
Seasonal ATN	Equation 11	Equation 12	Equation 14	0	0
Seasonal K	Equation 11	Constant	Constant	N/A	N/A
Seasonal r	Constant	Equation 12	Constant	N/A	0
Seasonal x	Constant	Constant	Equation 14	0	N/A
Seasonal ATN −2°C	Equation 11	Equation 12	Equation 14	−2°C	0
Seasonal ATN −1°C	Equation 11	Equation 12	Equation 14	−1°C	0
Seasonal ATN +1°C	Equation 11	Equation 12	Equation 14	+1°C	0
Seasonal ATN +2°C	Equation 11	Equation 12	Equation 14	+2°C	0
Seasonal ATN T + I	Equation 11	Equation 12	Equation 14	+2°C	−50 Wm^−2^
Seasonal ATN GradT	Equation 11	Equation 12	Equation 14	0°C… + 3.7°C	0
Seasonal ATN −50 Wm^−2^	Equation 11	Equation 12	Equation 14	0	−50 Wm^−2^
Seasonal ATN −25 Wm^−2^	Equation 11	Equation 12	Equation 14	0	−25 Wm^−2^
Seasonal ATN +25 Wm^−2^	Equation 11	Equation 12	Equation 14	0	25 Wm^−2^
Seasonal ATN +50 Wm^−2^	Equation 11	Equation 12	Equation 14	0	50 Wm^−2^

First, we evaluated how seasonal changes in carrying capacity of primary producers affect ATN model dynamics, using an adjusted version of K described in Boit et al. (2012):

(11) $$K_{s}\left(t\right)=K_{c}\left(1+\frac{1}{4}\left(\frac{2}{1+e^{z\left(t-t_{\max}\right)}}-1\right)\right)$$

where $K_{c}=540,000\;\mathrm{\mu gC}/m^{3}$ is the original constant carrying capacity, $z=0.1\;{\mathrm{day}}^{-1}$ is a decay component and $t_{\max}=120\;\text{days}$ equaling to the middle of the 240‐day growing season (Figure 2a). This abiotic forcing simulates high primary production capacity when nutrients are highly available during the early‐season water column mixing period, followed by decreasing production capacity due to increasing nutrient limitation toward the mid‐growing season stratification period. By adjusting the seasonal carrying capacity model of Boit et al. (2012) this way, we can interpret the constant carrying capacity model as the average of the seasonally changing carrying capacity model and thus be better able to compare the two models (Figure 2a, Table 1).

Second, we evaluated the effect of seasonal changes in primary producers' growth on ATN model dynamics by multiplying the constant growth rate $r_{i}$ with a time‐varying light coefficient $c_{L}\left(t\right)$, that is, $r_{s,i}\left(t\right)=r_{i}c_{L}\left(t\right)$ where

(12) $$c_{L}\left(t\right)=\frac{c_{L,0}}{\mathrm{\lambda h}}\log\left(\frac{I_{0}+I\left(t\right)}{I_{0}+I\left(t\right)e^{-\mathrm{\lambda h}}}\right)$$

The half‐saturation constant for irradiance, $I_{0}=46\;{\mathrm{Wm}}^{-2}$, was set to 20% of the maximum irradiance (Wallace et al., 1996), whereas h=20m is the epilimnion depth and $\lambda =0.1\;m^{-1}$ is a typical value for the bulk attenuation coefficient (Wallace et al., 1996). The scaling factor $c_{L,0}=1.813$ was chosen so that average yearly total producer biomass was equal to the average total producer biomass of the constant model (Figure 2b, Table 1). The irradiance $I\left(t\right)$ at time t is expressed in units of Wm^−2^ and modeled as a half‐sine function which gives the maximum amount of radiation in the middle of the growing season and less toward the beginning and end of the season, thus mimicking the seasonally changing day length (Figure 2b):

(13) $$I\left(t\right)=I_{\mathrm{adj}}+150\;Wm^{-2}+80∙\left(2∙\sin\left(\frac{\mathrm{\pi t}}{t_{\mathrm{end}}}\right)-1\right)\;Wm^{-2}$$

Here, $I_{\mathrm{adj}}$ ($Wm^{-2}$) was used to adjust the mean irradiance level in the simulations.

Third, we evaluated how abiotic forcing of higher trophic levels influences ATN model dynamics by multiplying the consumers' constant metabolic rate $x_{i}$ with a time‐varying temperature‐dependent coefficient $c_{T}\left(t\right)$, that is, $x_{s,i}\left(t\right)=x_{i}c_{T}\left(t\right)$ where

(14) $$c_{T}\left(t\right)=c_{T,0}{Q_{10}}^{\left(T\left(t\right)-T_{0}\right)/10}$$

Here, $T_{0}=12{}^{\circ}C$ is the standard temperature and $Q_{10}=3$ is a temperature‐dependency coefficient (Boit et al., 2012). The time‐varying temperature $T\left(t\right)$ was modeled as a half‐sine function to have the warmest temperature in the middle and colder temperatures at the beginning and the end of the growing season (Figure 2c):

(15) $$T\left(t\right)=T_{0}+5\left(2∙\sin\left(\frac{\mathrm{\pi t}}{t_{\mathrm{end}}}\right)-1\right){}^{\circ}C+T_{\mathrm{adj}}$$

where $T_{\mathrm{adj}}$ is a free parameter used to make small adjustments to the average temperature. With $T_{\mathrm{adj}}=0$°C, the $T\left(t\right)$ temperatures range between 7°C and 17°C. The $c_{T}\left(t\right)$ used by Boit et al. (2012) produces $x_{s,i}\left(t\right)$ values typically exceeding the seasonally invariant $x_{i}$. Hence, to make the model outputs more comparable, we added a scaling coefficient $c_{T,0}=0.84$, which was chosen again so that the yearly average total producer biomass equals the yearly average total producer biomass of the constant model.

Finally, by using the ATN model configuration where all three modeled abiotic drivers are set to follow a seasonal pattern (Table 1, Figure 2), we evaluated how adjustment of the average temperature $T\left(t\right)$ and irradiance $I\left(t\right)$, which affect the mass‐specific metabolic rate of consumers $x_{i}$ and the light‐dependent growth rate of primary producers $r_{i}$, respectively, influences ATN model dynamics. For this, the average temperature was decreased or increased by 1°C and 2°C (*T*_adj_ = ±1°C, ±2°C), whereas the average irradiance was decreased or increased by 25 Wm^−2^ or 50 Wm^−2^ ($I_{\mathrm{adj}}=\pm 25\;{\mathrm{Wm}}^{-2},\pm 50\;{\mathrm{Wm}}^{-2}$). To test for potential interacting effects of increased temperature and decreased light availability associated with predicted impacts of ongoing global warming and brownification of freshwater ecosystems (Blanchet et al., 2022; Woolway et al., 2020), we run the ATN model after increasing T by 2°C—which is a conservative estimate of the average temperature increase of summer surface waters in large Austrian lakes by 2050 (Dokulil, 2014)—and decreasing I by 50 Wm^−2^. We also evaluated how a gradual 0.037°C year^−1^ increase in water temperature as observed in LC (Adrian et al., 2009) influences productivity of primary producers and consumption gains of consumers (see “gain from resources (*j*)” in equation 2), thereby simulating the effects of warming climate on food‐web productivity. We simulated this by incrementing $T_{\mathrm{adj}}$ by 0.037°C each year for 100 years, starting from $T_{\mathrm{adj}}=0{}^{\circ}C$ and ending up with $T_{\mathrm{adj}}=3.7$°C.

### 2.3 Visualization of simulation results

The effects of the inclusion of temporal variation in carrying capacity (K) and mass‐specific growth rates of primary producers ($r_{i}$) and metabolic rates of consumers ($x_{i}$) on ATN model dynamics were illustrated for the following trophic guild groups (see Table S1 for guild abbreviations): (i) phytoplankton (Alg1–5, APP), (ii) ciliates (Cil1–5), (iii) rotifers (Rot1–3, Asp), (iv) herbivorous crustaceans (Cru), (v) carnivorous crustaceans (Cyc, Lep), (vi) larval and juvenile whitefish (Whi0–1), (vii) adult whitefish (Whi2–4), (viii) larval and juvenile perch (Per0–1), and (ix) adult perch (Per2–4). For each ATN model configuration (Table 1), the biomasses and productivities of each guild were simulated over 240 years (240‐day long growing seasons), but the first 150 years were omitted to allow the system to settle into its dynamic equilibrium.

The effects of ATN model configuration and adjusted average values of temperature and irradiance on seasonal (i.e., within‐year) biomass dynamics (Table 1, Figure 2) were visualized for each trophic guild group as relative biomass differences $\Delta B_{\mathrm{rel}}(t)$ over the last simulation year (year 250):

(16) $$\Delta B_{\mathrm{rel}}\left(t\right)=\frac{B\left(t\right)-B\left(t_{\mathrm{init}}\right)}{B\left(t_{\mathrm{init}}\right)}$$

This allows direct comparison of the relative effects of ATN model configuration on the degree and timing of seasonal biomass development. Second, the effects of ATN model configuration (Table 1) on the relative standing stock biomasses and productivities of trophic guild groups were calculated and visualized. Standing stock biomasses of different guilds were measured as average biomasses calculated over the last 100 simulation years. While standing stock biomasses reflect the amount of carbon retained in each trophic guild group (determined by the balance of biomass gain through photosynthesis or consumption of prey guilds and loss through consumption by predator guilds; see equations 1, 2 and 7), the productivity reveals how much carbon is taken up by each consumer guild, thus providing a proxy for biomass flow in LC food web. The productivity of each consumer guild group was measured as a sum of consumption gains (see “gain from resources (*j*)” in equation 2) in the last 100 simulation years. The standing stock biomasses and productivities were further standardized by dividing the trophic guild group‐specific mean biomasses and sum of consumption gains by the sum of biomasses and gains over all studied guilds. These standardized measures of standing stock biomasses and productivities allow direct visual comparison of simulated biomass distribution across different trophic levels (functional guild groups) and of energy flow patterns in LC food web depending on the ATN model configuration (Table 1).

## 3 RESULTS

### 3.1 Seasonal biomass dynamics

**FIGURE 3:**
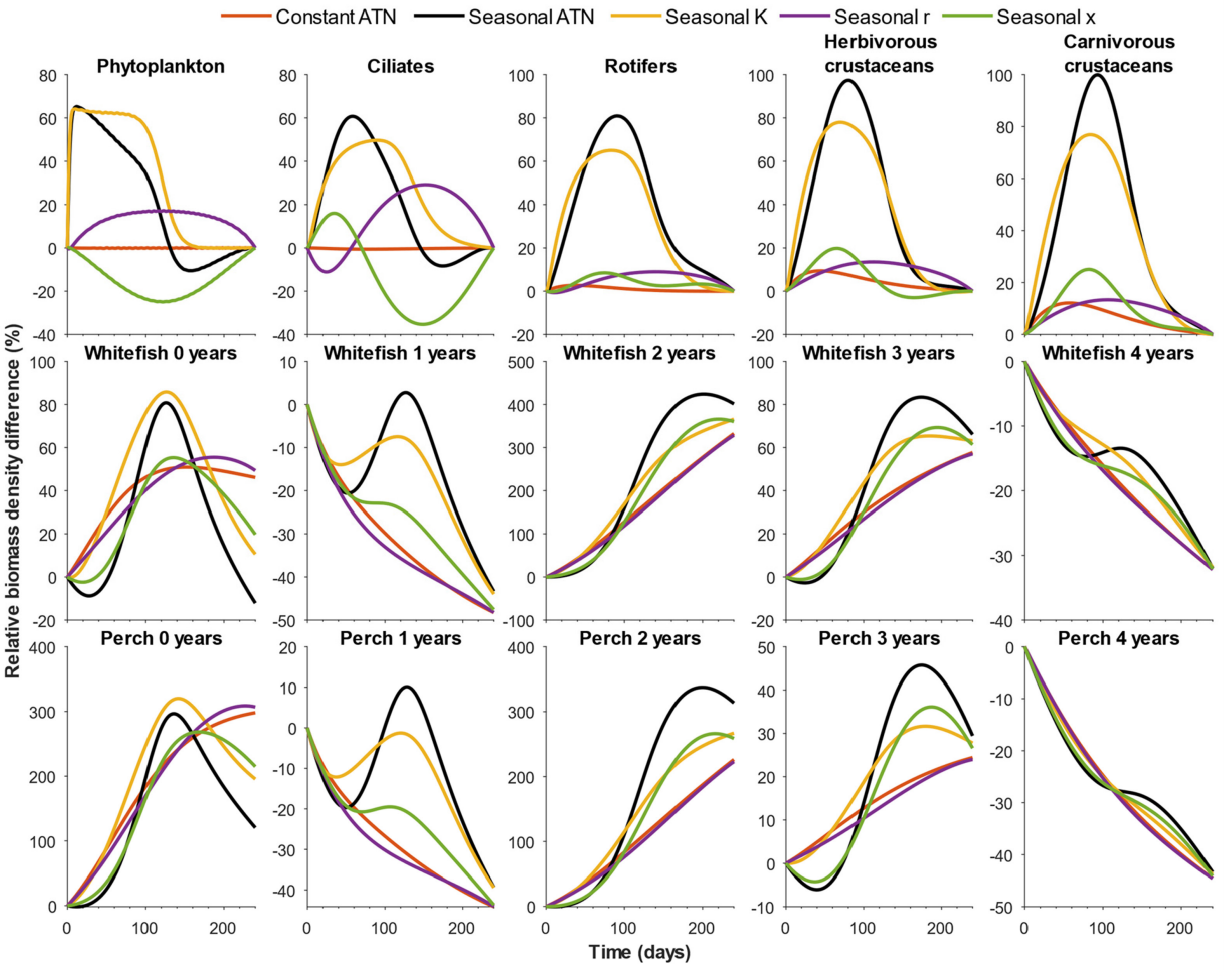
Seasonal biomass development (i.e., relative biomass density difference from the start of the growing season in the last simulation year) of the main trophic guilds simulated using different ATN‐model configurations (Table 1). Abbreviations: *K* = carrying capacity of primary producers, *r* = light‐dependent growth rate of primary producers, and *xx* = temperature‐dependent mass‐specific metabolic rate of consumers. Seasonal ATN refers to the model configuration where all three abiotic parameters are set to follow the given seasonal patterns (Figure 2, Table 1).

Inclusion of seasonal variation in the K, $r_{i}$, and $x_{i}$ parameters in the ATN model strongly influenced the simulated seasonal patterns in biomass development of primary producers and consumers in the LC food web (Figure 3). While using constant K, $r_{i}$, and $x_{i}$ parameters produced nearly seasonally invariable biomasses, the seasonally varying K (following a reverse sigmoid curve; Figure 2a) induced ca. 50–100% increase in biomass of phytoplankton, ciliates, and rotifers as well as of herbivorous and carnivorous crustaceans, associated with the simulated high carrying capacity of primary producers early in the growing season. For these functional groups, the inclusion of seasonally varying hump‐shaped $r_{i}$ and $x_{i}$ (Figure 2b,c) had somewhat opposite impacts on simulated biomass dynamics, with varying $r_{i}$ inducing an increased biomass maximum, whereas $x_{i}$ caused a deeper biomass minimum for phytoplankton and ciliates in mid‐ and late‐growing season, respectively (Figure 3).

The simultaneous inclusion of seasonal variation in K, $r_{i}$, and $x_{i}$ parameters in the ATN model induced an early‐season biomass boost for phytoplankton and ciliates, followed by a late‐season biomass bust associated with the declining K and heavy consumption by higher trophic levels. For rotifers, herbivorous and carnivorous crustaceans, the seasonally varying K, $r_{i}$, and $x_{i}$ parameters induced a broad biomass peak from early to middle growing season. For fish, seasonal changes in these ATN model parameters introduced a hump‐shaped biomass peak of 0‐year‐old whitefish and perch in the mid‐growing season, whereas 2‐ and 3‐year‐old fish showed an increased biomass peak later in the growing season (Figure 3). The contrasting patterns in simulated seasonal biomass dynamics arise from the fact that 0‐ and 1‐year‐old fish are heavily consumed by older perch (thus biomass declines toward late growing season), unlike the larger 2‐ and 3‐year‐old fish that are not predated (thus biomass increases toward the late growing season; Figure 3). The ≥4‐year‐old fish, including the 3‐year‐old fish from the previous year, are already at their maximum carrying capacity at the beginning of the growing season, therefore showing a drastic biomass decline over the growing season.

**FIGURE 4:**
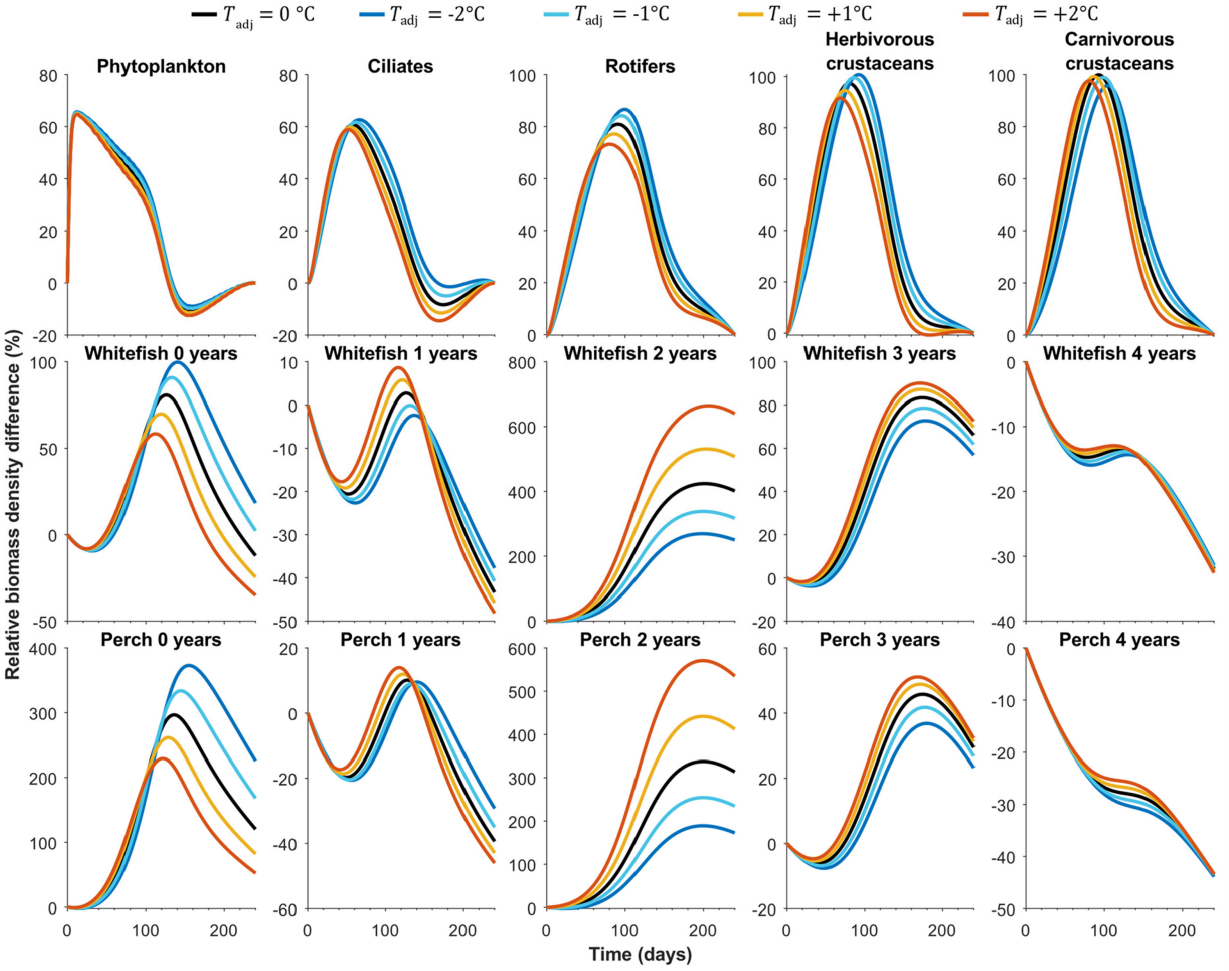
Seasonal biomass development (i.e., relative biomass density difference from the start of the growing season in the last simulation year) of the main trophic guilds simulated using the seasonal ATN model with adjusted average temperature ($T_{\mathrm{adj}}$) (Table 1).

Adjustment of the average temperature (parameter of the seasonal metabolic rate model; Table 1, Figure 2c) in the ATN model configuration had marked impacts on the simulated seasonal biomass development of fish guilds. While the simulated temperature increase of +1°C or +2°C slightly reduced the biomass of 0‐year‐old fish with a high metabolic rate and high consumption by older perch (Table S1), it drastically increased the biomass of 2‐year‐old whitefish and perch, and less of 1‐ and 3‐year‐old fish (Figure 4). In contrast, adjustment of the average irradiance (parameter of seasonal primary producer growth rate model) in the ATN model configuration (Table 1, Figure 2b) had only minor effects on the seasonal biomass dynamics of producers and consumers (Figure S1).

### 3.2 Biomass distribution and productivity among trophic guilds

**FIGURE 5:**
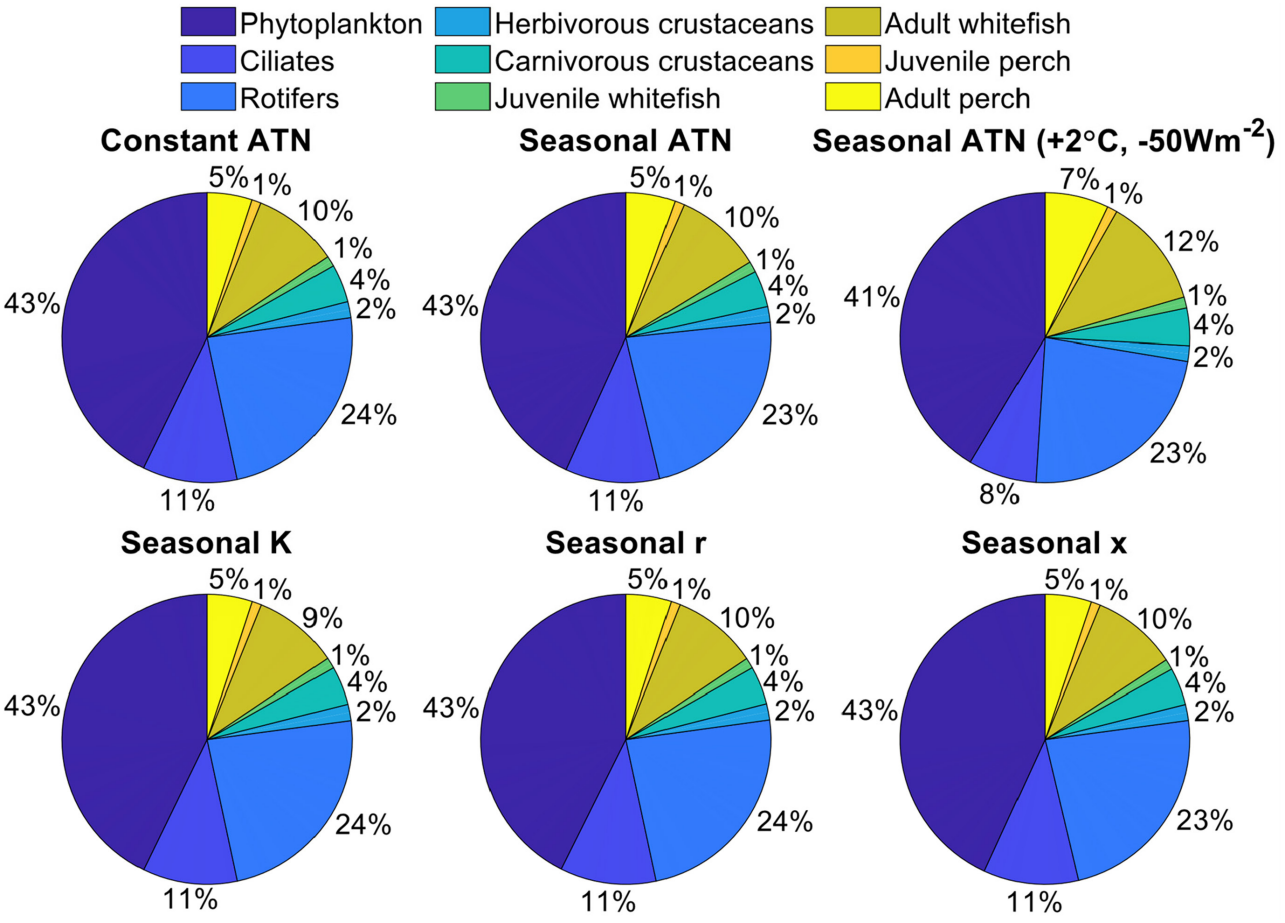
Relative (%) biomass distribution among producer (Phytoplankton) and consumer guild groups depending on the ATN model configuration (Table 1). The results are based on ATN simulations where all or one of the following parameters are set either as a constant value or they follow a seasonal pattern (Figure 2, Table 1): K = carrying capacity of primary producers, $r_{i}$ = light‐dependent growth rate of primary producers, and $x_{i}$ = temperature‐dependent mass‐specific metabolic rate of consumers. Seasonal ATN model configuration (Table 1) in the top right corner refers to a model where the average irradiance (equation 8) is reduced by 50 W m^−2^ to simulate reduced light availability and the average temperature is increased by 2°C to simulate warming effect.

**FIGURE 6:**
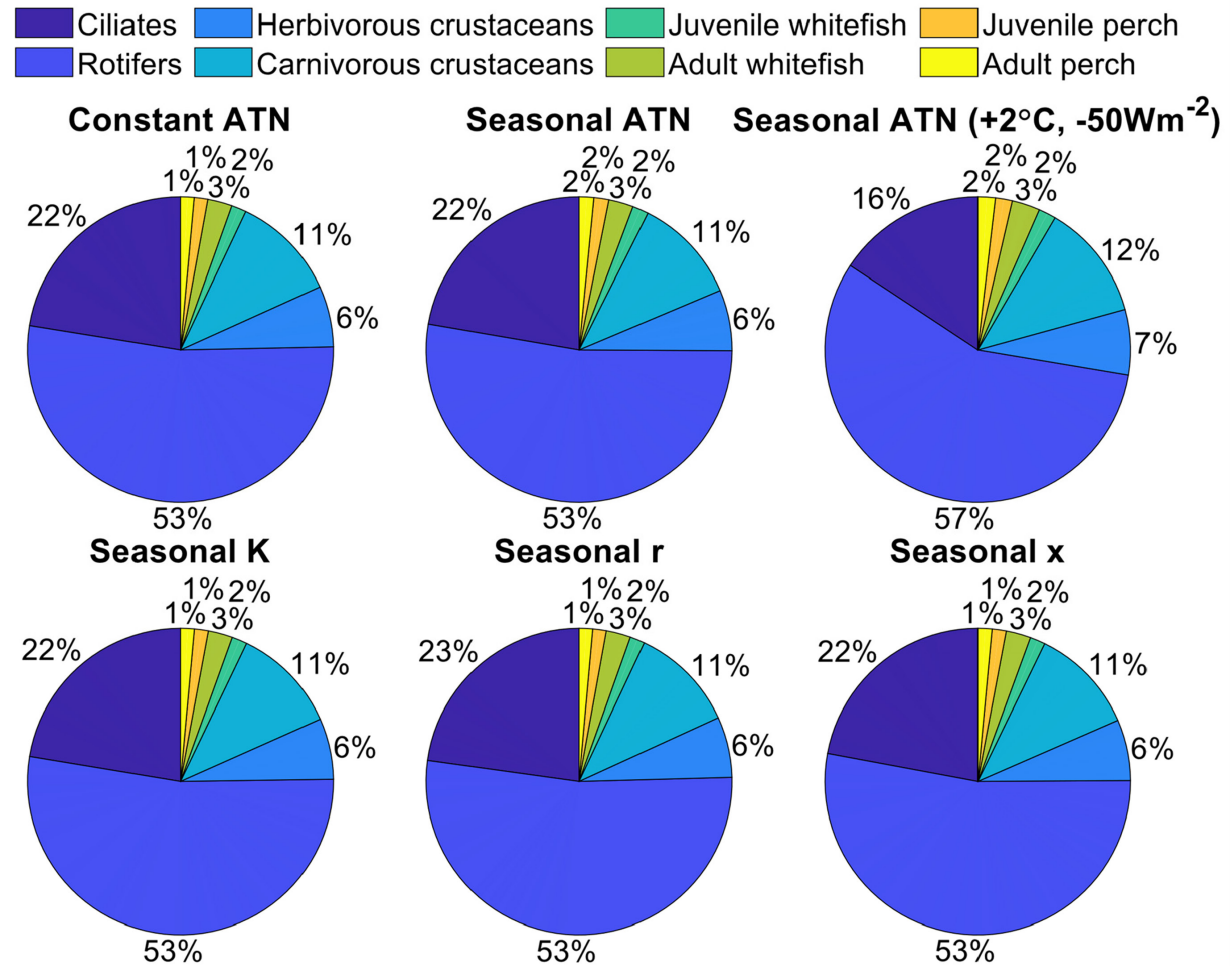
Relative (%) consumption gains (“productivity”) of consumer groups depending on the ATN model configuration. The results are based on ATN simulations where all or one of the following parameters are either set to a constant value or they follow a seasonal pattern (cf. Figure 1): K = carrying capacity of primary producers, $r_{i}$ = light‐dependent growth rate of primary producers, and $x_{i}$ = temperature‐dependent mass‐specific metabolic rate of consumers. Seasonal ATN model configuration (Table 1) in the top right corner refers to a model where the average irradiance (equation 8) is reduced by 50 W m^−2^ to simulate reduced light availability and the average temperature is increased by 2°C to simulate warming effect.

Despite the effects on seasonal biomass dynamics illustrated for the last simulation year, the ATN model configuration had no marked large‐scale effects on distribution of standing stock biomass among trophic levels (Figure 5) or on relative productivity (i.e., consumption gains) of consumer guilds (Figure 6). However, simultaneous adjustment of the average temperature (+2°C) and irradiance (−50 Wm^−2^) had more evident effects on biomass distribution (Figure 5) and consumer productivity (Figure 6) by reducing the relative biomass and production of ciliates but increasing the relative biomass of adult fish and the productivity of rotifers. These shifts are associated with the reduced productivity of light‐dependent primary producers (phytoplankton), which reduces the relative biomass of herbivorous ciliates. These primary consumers are, in turn, heavily predated by secondary consumers (i.e., large rotifers, cladocerans, and cyclopoids; Figure 5, Table S1) under increasing metabolic rate in warmer temperatures, which further support increasing relative biomass accumulation to top predator fish. Despite some effects on standing stock biomasses and productivity, the biomass (carbon) flows in the food web, illustrated as proportional consumption gains, were nearly constant regardless of the ATN model configuration (Figure S2, Table 1).

### 3.3 Effect of gradual temperature increase

**FIGURE 7:**
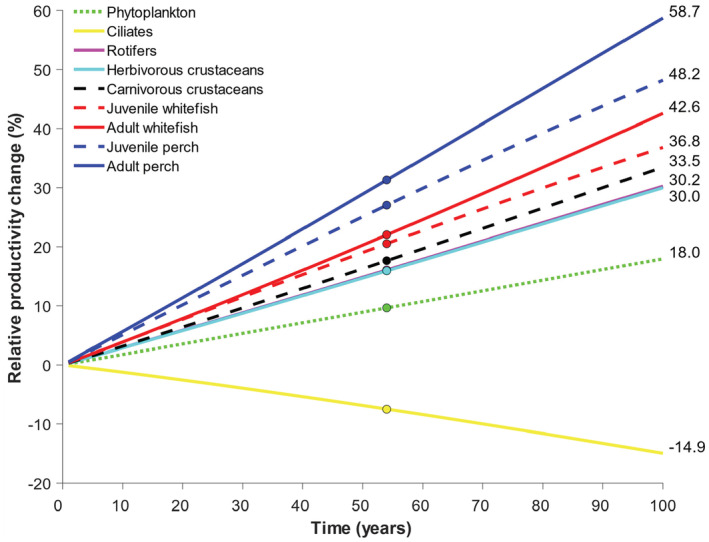
Relative changes (%) in the productivity of primary producers (i.e., phytoplankton) and consumption gains of different consumer guilds along a simulated gradual temperature increase of 0.037°C year^−1^ observed in Lake Constance (Adrian et al., 2009). The points indicate the times equalling to a simulated fixed temperature increase of 2°C.

The simulated gradual temperature increase of 0.037°C year^−1^ observed in Lake Constance (cf. Adrian et al., 2009) changed the ATN model dynamics so that the relative productivity (i.e., consumption gains) of all consumer guild groups increased, except that of ciliates (Figure 7). While the simulated phytoplankton productivity increased by 18%, the consumption gains of rotifers, herbivorous and carnivorous crustaceans increased by approx. 30% during the 100‐year simulation period. The juvenile and adult whitefish and perch showed approx. 40–60% increase in consumption gains during the 100‐year simulation period, indicating that the adjusted ATN model predicts cumulative positive effect of gradual temperature increase on the highest consumer levels.

## 4 DISCUSSION

Allometric trophic network (ATN) models have many theoretical and applied applications (summarized by Martinez, 2020), including simulation of community‐level responses to fisheries (Kuparinen et al., 2016) and to environmental stochasticity (Kuparinen et al., 2019). However, the influence of abiotic forcing on ATN model dynamics has remained largely unexplored, mainly because many ATN studies have rather focused on theoretical analyses across randomly generated food webs as opposed to specific study systems. Here, we mechanistically integrated abiotic drivers to the consumer–resource dynamics described by the ATN model for the Lake Constance food web (Figures 1 and 2, Table 1). The ATN model simulations demonstrated contrasting impacts of different abiotic drivers on the main functional groups. In general, adding seasonal variation in the producer carrying capacity (K) had stronger impacts on seasonal biomass development of primary producers and invertebrate consumers as compared to temperature‐dependent mass‐specific metabolic rate of consumers ($x_{i}$) or seasonal light‐dependent growth rate of primary producers ($r_{i}$) (Figure 3). The simulated effect of abiotic forcing on seasonal biomass development diminished toward the highest consumers, that is, adult fish. While adjustment of the average irradiance had minor effects (Figure S1), increasing average temperature by +1°C or +2°C in the seasonally varying ATN model suppressed the seasonal biomass peak of 0‐year‐old fish but increased the biomass peaks of adult (especially 2‐year‐old) perch and whitefish (Figure 4). Yet, the overall effect of ATN model configuration (Table 1) on simulated standing stock biomasses (Figure 5) and productivities (consumption gains; Figure 6) was minor, indicating that the ATN model developed for the pelagic Lake Constance food web is relatively insensitive to the adjustment of abiotic K, $x_{i}$ and $r_{i}$ parameters.

### 4.1 Abiotic forcing of seasonal biomass dynamics

Boit et al. (2012) found that adding minimal abiotic forcing markedly improved the ATN model fit with empirical data of seasonal dynamics and size‐abundance distribution of the phytoplankton community in Lake Constance. This implies that ATN models should likely consider seasonal variation in some key parameters determining the biomass accumulation and transfer from producers up to top predators. We took the next step in analyzing potential impacts of abiotic forcing in ATN model dynamics by looking separately at the responses of different trophic guild groups, including age‐structured fish populations (cf. Kuparinen et al., 2016), to seasonally varying carrying capacity (K) and light‐dependent growth rate ($r_{i}$) of primary producers and temperature‐dependent mass‐specific metabolic rate of consumers ($x_{i}$) (Figure 2, Table 1). We expected temporal changes in K and $x_{i}$ to be more important drivers of within‐year biomass dynamics than the light‐dependent $r_{i}$, because the former set the basal limits for secondary production and influence all consumer guilds, respectively. Adding seasonal variation in these key parameters, especially in K and $x_{i}$, induced seasonality in biomass development, contrary to the dynamics of an ATN model where these parameters were constants, resulting in nearly constant seasonal biomasses of primary producers and invertebrate consumers (Figure 3). Including a seasonal decline in K induced the development of a phytoplankton biomass peak early in the growing season, followed by a biomass peak of herbivorous and carnivorous pelagic invertebrates, a pattern observed also empirically in seasonal dynamics of LC food web (Gaedke et al., 2002). These primary and secondary consumers subsequently declined following the phytoplankton biomass decline in the mid‐growing season. Adding seasonal variation in $x_{i}$ induced a U‐shaped pattern in phytoplankton biomass development likely due to increased consumption by ciliates in early growing season, followed by intense grazing by rotifers and herbivorous crustaceans toward mid‐growing season when the consumer metabolic rates were at the highest level. Although here we did not use empirical abiotic or temporal community data to validate our simulations, our study suggests that using time‐dependent parameters in ATN models could better reflect the temporal nature of abiotic drivers modifying, for example, community dynamics, consumers' energetic demands, and seasonal changes in resource availability (e.g., Gårdmark & Huss, 2020; Kharouba & Wolkovich, 2020; McMeans et al., 2015).

### 4.2 Adjusted average temperature and irradiance

Climate change, together with intensive land use (e.g., agriculture and forestry), is predicted to increase surface water temperatures (Gobiet et al., 2014), harmful algal blooms (Elliott, 2012), and loading of nutrients and colored dissolved organic carbon into lakes (Blanchet et al., 2022; Karlsson et al., 2009; Kritzberg et al., 2019). While higher surface water temperatures may increase the metabolic rate of consumers (Lindmark et al., 2017; Sheridan & Bickford, 2011) and reduce nutrient and oxygen availability due to impaired water column mixing (Woolway et al., 2020; Yankova et al., 2017), changes in light availability associated with, for example, browning have also been shown to influence the growth and biomass of producers and consumers (Blanchet et al., 2022; Karlsson et al., 2009; van Dorst et al., 2020). We modified the abiotic forcing parameters developed by Boit et al. (2012) to test how the inclusion of seasonality associated with a change in mean temperature and irradiance affect biomass dynamics in LC food web. Adjustment of the average irradiance had virtually no effect (Figure S1), and the temperature adjustment had only minor effect on seasonal biomass development of the lowest (primary producers and consumers) and highest (≥4‐year‐old fish) trophic levels (Figure 4). In contrast, a temperature decrease of 1–2°C increased the biomass peaks of 0‐year‐old fish, whereas a temperature increase of 1–2°C increased the biomass peaks of 2‐year‐old fish and to a lesser extent of 1‐ and 3‐year‐old fish (Figure 4). The observed warming‐induced decline of 0‐year‐old fish and increase of adult fish results from increased predation pressure on fish larvae associated with increased metabolic and thus consumption rates of large fish.

We also found a gradual temperature increase of 0.037°C year^−1^ (as observed in Lake Constance; Adrian et al., 2009) to increase the relative productivity of producers by 18% but decrease that of ciliates by ca. 15% (Figure 7). The ATN model simulations suggested increased productivity for all higher consumer guild groups, including invertebrates (ca. +30%), larval and juvenile stages of fish (ca. +35–50%) and adult fish (ca. +40–60%). The negative effect of gradual temperature increase on the productivity of herbivorous ciliates was likely associated with their relatively high metabolic rate and high consumption by several predatory invertebrates, including large ciliates, rotifers, cladocerans, and copepods (Table S1). A previous modeling study of LC food web suggests that no strong phenological mismatches in consumer–resource interactions should be expected with seasonally homogenous warming, but only when warming will be seasonally heterogeneous (Straile et al., 2015). Therefore, future studies could test whether the dynamics of ATN simulations would depend more on the timing (e.g., peaks in temperature and irradiance) than on the degree of abiotic forcing.

In our study, adjustment of the average irradiance had only minor effects on the seasonal biomass dynamics of the main trophic guilds in LC food web (Figure S1). Following largely Boit et al. (2012) approach, we simulated seasonal changes in light availability by adjusting the producer growth rate $r_{i}$ with a light coefficient $\;c_{L}\left(t\right)$ based on simulated irradiance $I\left(t\right)$ at a given day during the growing season (Figure 2, Table 1). However, this adjustment of average irradiance evidently caused only minor effects on the seasonal development of phytoplankton biomass and even less of different consumer guilds (Figure S1). In nature, phytoplankton taxa show marked differences in light utilization efficiency, with harmful (toxin‐producing) and nonedible cyanobacteria being particularly adapted to low‐light conditions and green, more edible algae being adapted to higher light environments (e.g., Schwaderer et al., 2011). Such differences in light utilization efficiency among nonedible and edible phytoplankton taxa could be accounted for in future development of abiotic forcing in ATN models. Moreover, while our simulations of light availability effects on producer growth rate could indirectly influence consumer biomass and consumption gains, in nature light conditions can have strong direct impacts on feeding efficiency and thus growth of visual predators (e.g., van Dorst et al., 2020).

### 4.3 Biomass distribution and productivity among trophic guilds

We expected the long‐term mean standing stock biomasses and productivity of different trophic guilds simulated over 100 years to be relatively unresponsive to within‐year variation in abiotic drivers. However, our ATN model simulations suggest that simultaneous warming and reduced light availability may induce a shift toward a slightly top‐heavier food web in LC. Introducing seasonal variation only in the K or $x_{i}$ parameters in the ATN model had no effect on the simulated distribution of standing stock biomass and productivity (i.e., consumption gains) across trophic guilds, whereas seasonality in the light‐dependent growth rate of primary producers ($r_{i}$) slightly increased the simulated biomass and productivity of rotifers. A more evident shift toward a top‐heavy food web was observed when all three abiotic drivers showed seasonal variation and the average temperature was simultaneously increased by +2°C and the average irradiance was decreased by −50 Wm^2^, simulating environmental changes associated with global warming and reduced light availability due to browning or eutrophication. Hence, our results indicate that the abiotic drivers can have contrasting effects on the ATN model dynamics when used individually or in combination, as well as depending on the average value of the simulated abiotic parameters. While our findings contradict some modeling studies of warming and eutrophication impacts on food webs (Binzer et al., 2012, 2016), they are partly supported by experimental studies indicating reduced producer but increased consumer biomass with warming (Shurin et al., 2012), particularly in environments where plentiful nutrients lead to increased biomass of higher trophic levels and strong top‐down control of producer biomass (O'Connor et al., 2009). Although we found some support for altered biomass distribution among trophic guilds, the impacts of abiotic forcing were generally minor (Figure 5). Thus, in terms of large‐scale biomass dynamics (i.e., mean biomass distribution among trophic levels simulated across 100 years), our ATN model for LC food web is evidently not sensitive to seasonally varying abiotic drivers. However, it should be noted that the model does not effectively account for potential seasonal or annual fluctuations in nutrient availability, which can be among the major drivers of bottom‐up and top‐down control in lake communities (Rogers et al., 2020) and thus should likely be incorporated in future ATN model developments.

### 4.4 Study limitations

Our study aimed at testing the effects of seasonally varying abiotic drivers on ATN model dynamics. In general, the results indicate minor effects of seasonally varying abiotic drivers on biomass accumulation and transfer across main guild groups in the pelagic LC food web. Naturally, our findings are limited to one food web, but at the same time finely resolved complex lake food webs remain rare, particularly those that include realistic life‐history structuring. Use of empirical or randomly generated data of environmental drivers in the ATN simulations, followed by comparison of simulation results with empirical data of community dynamics, would confirm the ATN model applicability to simulation and prediction of natural community‐ and ecosystem‐level processes (cf. Boit et al., 2012), which could then support ecosystem‐based environmental management. To test for the generality of food‐web responses, abiotic forcing should also be accounted and tested for by using ATN models parametrized for other ecosystems or using random networks (cf. Williams & Martinez, 2000). Moreover, while ATN models simulate biomass transfer and accumulation in food webs, stoichiometry (e.g., C:N:P balance) and food quality (e.g., fatty acid composition and quantity) are fundamental factors affecting trophic transfer efficiency as well as growth, survival, and fitness of individuals, which ultimately modify community‐level responses to abiotic drivers in natural ecosystems (Glibert, 2012; Sardans et al., 2012; Twining et al., 2015).

## 5 CONCLUSIONS

Our study demonstrates the potential of using time‐dependent parameters (reflecting seasonal changes in abiotic drivers) in ATN models to better reflect temporal fluctuations in community dynamics. When it comes to the long‐term dynamics and biomass distribution among trophic guilds, our simulations suggest that the developed ATN model for pelagic LC food web is relatively insensitive to the adjustment of the abiotic drivers that were originally incorporated by Boit et al. (2012). However, it should be noted that abiotic drivers show larger, more random fluctuations in nature than those simulated in our study. More research is needed to reveal how, for example, the timing, magnitude, and frequency of fluctuations in abiotic drivers may shape the simulation outcomes, preferably using ATN models developed for contrasting communities. Such mechanistic models considering abiotic drivers of food‐web dynamics are highly needed for sound management and mitigation actions in aquatic ecosystems influenced by multiple human stressors (Kovalenko, 2019; Woodward et al., 2010).

## AUTHOR CONTRIBUTIONS

**Antti P. Eloranta:** Conceptualization (equal); formal analysis (equal); investigation (equal); visualization (equal); writing – original draft (lead); writing – review and editing (lead). **Tommi Perälä:** Conceptualization (equal); formal analysis (equal); investigation (equal); methodology (lead); visualization (equal); writing – original draft (equal); writing – review and editing (equal). **Anna Kuparinen:** Conceptualization (equal); formal analysis (supporting); funding acquisition (lead); investigation (supporting); project administration (lead); resources (lead); supervision (lead); writing – original draft (supporting); writing – review and editing (supporting).

## CONFLICT OF INTEREST STATEMENT

The authors have no conflicts of interest to declare. All co‐authors have seen and agree with the contents of the manuscript, and there is no financial interest to report. The submitted manuscript is original work and is not under review in any other journal.

## Supporting information


Appendix S1.

## Data Availability

This study does not include new empirical data that could be shared in public archives. However, the Matlab codes used for running the simulations are deposited in Dryad (https://doi.org/10.5061/dryad.g4f4qrfv7).
