# Supplementary material for: Effects of temporal abiotic drivers on the dynamics of an allometric trophic network model
Source: Ecol Evol. 2023 Mar 23;13(3):e9928. doi: 10.1002/ece3.9928 (PMC10034489; doi:10.1002/ece3.9928)
Supplement: Supplementary file 1 — Appendix S1. [file ECE3-13-e9928-s001.docx]

**Supporting Information**

**Figure S1**. Seasonal biomass development (i.e. relative biomass density difference from the start of the growing season in the last simulation year) of the main trophic guilds simulated using the seasonal ATN model with adjusted average adjusted irradiance (*I*_adj_) (Table 1).


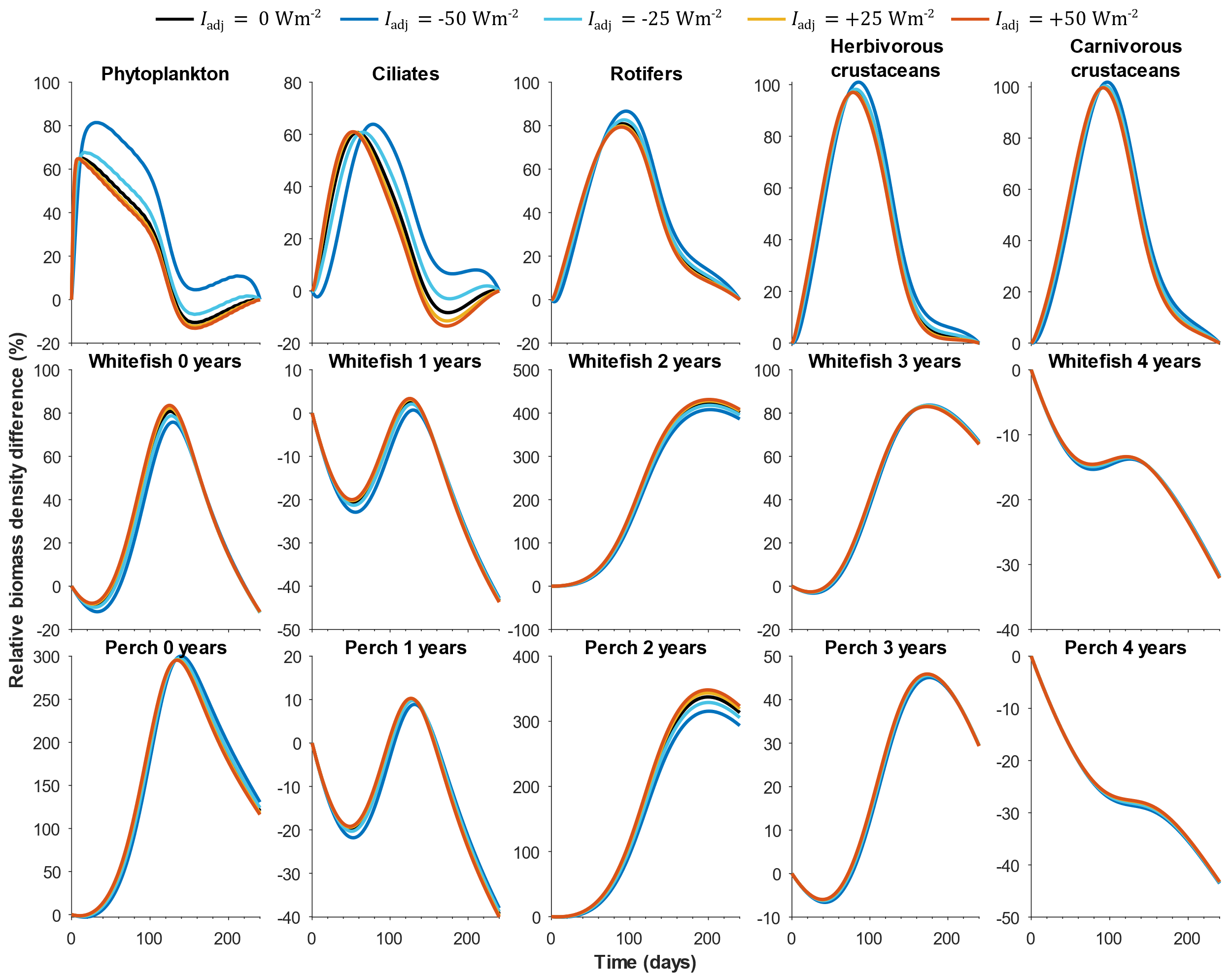


**Figure S2.** Consumption gain proportions (%) of different consumer guilds depending on ATN-model configuration (Table 1). Abbreviations: K = producer carrying capacity (*K*), r = light-dependent growth rate of producers ($r_{i}$), x = temperature-dependent mass-specific metabolic rate of consumers ($x_{i}$).


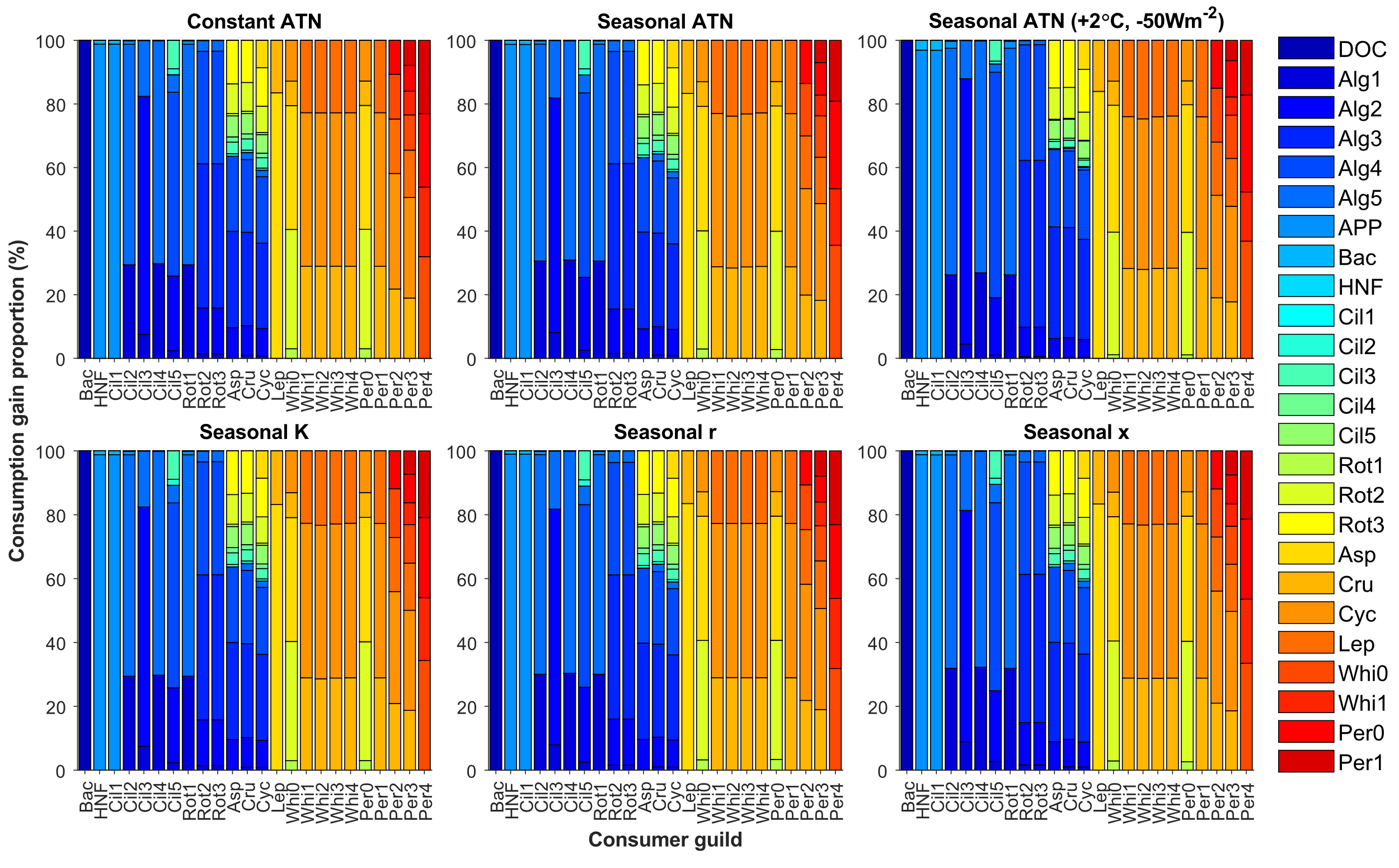


**Table S1** Lake Constance food web with size-related parameters and prey ranges (adapted from Boit et al. 2012; Kuparinen et al. 2016).

| **ID** | **Name** | **Description** | **Body mass^†^** | **x_i_**, **r_i_**^‡^ | **Diet ID^*^** |
| --- | --- | --- | --- | --- | --- |
| -1 /0 | POC/ DOC | Particulate / Dissolved organic carbon | n.a. | n.a. | n.a. |
| 1 | Alg1 | Single-cell algae | 6.40E-5 | 1 | n.a. |
| 2 | Alg2 | Large, single-cell algae or colonies | 2.56E-4 | 0.9 | n.a. |
| 3 | Alg3 | Filamentous blue and green algae | 3.20E-5 | 1.09 | n.a. |
| 4 | Alg4 | Diatoms, algal colonies | 1.28E-4 | 1 | n.a. |
| 5 | Alg5 | Small, coccal algae | 8.00E-6 | 1.2 | n.a. |
| 6 | APP | Autotrophic picoplankton | 2.50E-7 | 0.6 | n.a. |
| 7 | Bac | Heterotrophic bacteria | 1.56E-8 | 0.04 | 0 |
| 8 | HNF | Heterotrophic nanoflagellates, **B**^§^ | 8.00E-6 | 0.43 | 6–7 |
| 9 | Cil1 | Small ciliates, **B** | 2.56E-4 | 0.14 | 6–7 |
| 10 | Cil2 | Small ciliates, **B/H** | 2.05E-3 | 0.18 | 1, 5–8 |
| 11 | Cil3 | Medium-size ciliates, **H** | 4.10E-3 | 0.15 | 1–2, 5, 8 |
| 12 | Cil4 | Medium-size ciliates, **H** | 8.19E-3 | 0.15 | 1, 5, 8 |
| 13 | Cil5 | Larger ciliates, **O** | 6.55E-2 | 0.1 | 1–2, 4–5, 8–11 |
| 14 | Rot1 | Small rotifers, **B/H** | 1.64E-2 | 0.13 | 1, 5–8 |
| 15 | Rot2 | Medium-size rotifers, **H** | 3.28E-2 | 0.12 | 1–9 |
| 16 | Rot3 | Large rotifers, **O** | 6.55E-2 | 0.11 | 1–5, 8–9 |
| 17 | Asp | Large rotifers, **C** | 6.55E-2 | 0.12 | 2–4, 8–16 |
| 18 | Cru | Mostly cladocerans (daphnids), **H/O** | 8.39E+0 | 0.07 | 1–16 |
| 19 | Cyc | Cyclopoid copepods, **O/C** | 1.05E+0 | 0.07 | 1–5, 8–17 |
| 20 | Lep | Large, carnivorous cladocerans*,* **C** | 6.71E+1 | 0.04 | 17–18 |
| 21 | Whi0 | Whitefish larvae, **C** | 9.40E+2 | 0.0758 | 14–19 |
| 22 | Per0 | Perch larvae, **C** | 3.67E+2 | 0.0802 | 14–19 |
| 23 | Whi1 | 1-year-old whitefish juveniles, **C** | 1.83E+6 | 0.0559 | 18–20 |
| 24 | Per1 | 1-year-old perch juveniles, **C** | 1.08E+6 | 0.0596 | 18–20 |
| 25 | Whi2 | 2-year-old whitefish, **C** | 9.60E+6 | 0.0492 | 18–20 |
| 26 | Per2 | 2-year-old perch, **C** | 5.17E+6 | 0.0530 | 18–22 |
| 27 | Whi3 | 3-year-old whitefish, **C** | 2.26E+7 | 0.0458 | 18–20 |
| 28 | Per3 | 3-year-old perch, **C** | 1.11E+7 | 0.0497 | 18–24 |
| 29 | Whi4 | 4-year-old and older whitefish, **C** | 3.84E+7 | 0.0437 | 18–20 |
| 30 | Per4 | 4-year-old and older perch, **C** | 1.71E+7 | 0.0479 | 21–24 |

^†^in (*µgC*/individual).

**^‡^**relative producer growth rate $r$ and consumer and fish metabolic rate $x$ (1/day); scaling is done with respect to the growth rate of guild 1.

^*^ID of resource guild.

§ General diet description: B = bacterivore, H = herbivore, C = carnivore & O = omnivore.

**Table S2** Summary of the ATN model parameters for Lake Constance.

| **Parameter** | **Unit** | **Value** | **Description** | **Reference*** |
| --- | --- | --- | --- | --- |
| $K_{0}$ | μgC/m^3^ | 540000 | Producer carrying capacity | Boit et al. (2012) |
| $x_{i}$ | 1/day | 0.04–0.43 | Mass-specific metabolic rate^1^ | Brose et al. (2006) |
| $r_{i}$ | 1/day | 0.6–1.2 | Mass-specific growth rate for autotrophs^1^ | Brose et al. (2006) |
| $c_{ij}$ |  | 1, except 2 for $i=j$ | Producer competition coefficient | Uusi-Heikkilä et al. (2018) |
| $f_{a}$ |  | 0.2/0.4 | Activity metabolism coefficient (bacteria/others) | Boit et al. (2012), Uusi-Heikkilä et al. (2018) |
| $f_{m}$ |  | 0.1 | Maintenance respiration coefficient | Boit et al. (2012) |
| $y_{ij}$ |  | 4/8 | Maximum consumption rate scaling factor (ectotherm vertebrates/invertebrate predators) | Brose et al. (2006), Yodzis and Innes (1992) |
| $e_{ij}$ |  | 0.45/0.85 | Assimilation efficiency (plant/animal food) | Bland et al. (2019) |
| $d_{ij}$ | m^3^/μgC | 0–1 | Feeding interference coefficient | Skalski and Gilliam (2001), Bland et al. (2019)^2^ |
| $q$ |  | 1.2 | Functional response shape parameter | Boit et al. (2012) |
| $\omega_{ij}$ |  | 0–1 | Relative prey preference | Boit et al. (2012) |
| $s_{i}$ |  | 0.2 | Fraction of exudation | Boit et al. (2012) |
| $B0_{ij}$ | μgC/m^3^ | 1500–150000 | Half-saturation densities | Bland et al. (2019) ^2^ |

^1^ Relative rates with respect to guild 1; see Table S1.

^2^ For detritivores we used $e_{ij}=0.45$, $B0_{ij}=150000$ and $d_{ij}=1$.

* References listed in a separate reference list (“References Table S2”).

**References Table S2**

Bland, S., Valdovinos, F. S., Hutchings, J. A., & Kuparinen, A. 2019. The role of fish life histories in allometrically scaled food‐web dynamics. – Ecology and Evolution 9: 3651–3660.

Boit A., Martinez N.D., Williams R.J. & Gaedke U. 2012. Mechanistic theory and modelling of complex food-web dynamics in Lake Constance. – Ecology Letters 15: 594–602.

Brose U., Williams R.J. & Martinez N.D. 2006. Allometric scaling enhances stability in complex food webs. – Ecology Letters 9: 228–236.

Skalski G.T. & Gilliam J.F. 2001 Functional responses with predator interference: viable alternatives to the Holling Type II model. – Ecology 82: 3083–3092.

Uusi-Heikkilä, S., Perälä, T. & Kuparinen, A. 2018. Species’ ecological functionality alters the outcome of fish stocking success predicted by a food-web model. – Royal Society Open Science 5: 180465.

Yodzis P. & Innes S. 1992. Body size and consumer-resource dynamics. – American Naturalist 139: 1151–1175.
